# Naturally Killing the Silent Killer: NK Cell-Based Immunotherapy for Ovarian Cancer

**DOI:** 10.3389/fimmu.2019.01782

**Published:** 2019-08-09

**Authors:** Sarah Nersesian, Haley Glazebrook, Jay Toulany, Stephanie R. Grantham, Jeanette E. Boudreau

**Affiliations:** ^1^Department of Microbiology and Immunology, Dalhousie University, Halifax, NS, Canada; ^2^Department of Medicine, Dalhousie University, Halifax, NS, Canada; ^3^Department of Pathology, Dalhousie University, Halifax, NS, Canada

**Keywords:** natural killer cell, immunotherapy, ovarian cancer immunology, oncoimmunology, tumor microenvironment, high grade serous ovarian cancer

## Abstract

Ovarian cancer (OC) is diagnosed in ~22,000 women in the US each year and kills 14,000 of them. Often, patients are not diagnosed until the later stages of disease, when treatment options are limited, highlighting the urgent need for new and improved therapies for precise cancer control. An individual's immune function and interaction with tumor cells can be prognostic of the response to cancer treatment. Current emerging therapies for OC include immunotherapies, which use antibodies or drive T cell-mediated cancer recognition and elimination. In OC, these have been limited by adverse side effects and tumor characteristics including inter- and intra-tumoral heterogeneity, lack of targetable antigens, loss of tumor human leukocyte antigen expression, high levels of immunosuppressive factors, and insufficient immune cell trafficking. Natural killer (NK) cells may be ideal as primary or collateral effectors to these nascent immunotherapies. NK cells exhibit multiple functions that combat immune escape and tumor relapse: they kill targets and elicit inflammation through antigen-independent pathways and detect loss of HLA as a signal for activation. NK cells are efficient mediators of tumor immune surveillance and control, suppressed by the tumor microenvironment and rescued by immune checkpoint blockade. NK cells are regulated by a variety of activating and inhibitory receptors and already known to be central effectors across an array of existing therapies. In this article, we highlight interactions between NK cells and OC and their potential to change the immunosuppressive tumor microenvironment and participate in durable immune control of OC.

## Ovarian Cancer

Ovarian cancer (OC) is the leading cause of death from gynecologic malignancies with a 5-year survival of <50% (1). The majority of cases are diagnosed at advanced stages (III and IV), when treatment becomes especially challenging, earning OC its “silent killer” moniker. Beyond stage III, OC disseminates into the peritoneum, and patients present with bloating from a buildup of ascitic fluid in the abdomen (2). Current standard of care includes de-bulking surgery followed by a combination of platinum- and taxane-based chemotherapy. Despite these aggressive treatments, recurrence occurs in 60–70% of patients within 2–5 years; most will eventually succumb to OC (3).

Epithelial OC represents the majority of malignant ovarian tumors and arises from the epithelium of the ovary and fallopian tube. OC tumors are broadly divided into two subtypes: type I and II; these classifications carry both prognostic and predictive value (4). These subtypes are differentially responsive to the available treatments, and whether they may inform the development and application of more precise treatment, including immunotherapy, is the subject of active investigation. Type I OC includes low-grade serous, endometrioid, clear cell, and mucinous carcinomas that together account for ~30% of ovarian tumors (4, 5). This subset is typically genetically stable and slow-growing. By contrast, type II OC is genetically unstable, more aggressive and accounts for the majority of OC mortality (4, 5). This subtype exclusively includes high-grade serous carcinomas, represents 70% of all ovarian tumors, and will be the focus of this review (6–8).

## NK Cells

NK cells are lymphocytes that reside in the peripheral blood and are highly efficient anti-tumor effectors (9–11). They comprise 5–10% of circulating lymphocytes and kill target cells without previous sensitization. NK cells differentiate from the common lymphoid progenitor in the bone marrow, are most closely related to T cells, and have similar abilities to expel perforin and granzymes for direct target cell killing (12, 13). In addition, they signal for target cell apoptosis through Fas and TRAIL pathways and secrete pro-inflammatory cytokines including IFN-γ and TNF (12). NK cells can be characterized into an array of categories based on the presence and density of surface markers expressed with some studies reporting up to 30,000 in one individual (14). At a superficial level they can be categorized into two main populations based on CD16 and CD56–expression; with CD56bright/CD16– functioning to primarily produce cytokines in the circulating blood, and CD56dim/CD16+ performing cytotoxicity in the tissues (12).

Rather than direct detection of antigens through a germline-rearranged receptor (the domain of T and B cells), NK cells recognize putative targets based on their expression of stress-induced ligands, upregulated on the cell surface consequent to DNA damage and heat shock, and in response to stimulation by environmental factors, including cytokines and chemokines (15–17). To avoid unwanted auto-aggression, NK cells are also sensitive to inhibition by “self” human leukocyte antigen (HLA) class I molecules; it is the net outcome of incoming activating and inhibitory signals that determines the NK cell response to a putative target (12). A complete summary of the receptors and cytokines produced by NK cells is beyond the scope of this review and can be found elsewhere (18–20). Here, we limit our discussion to those identified as relevant in OC, summarized in Table 1.

**Table 1:** NK cell receptors and their relevance to ovarian cancer.

**Receptor**	**Ligand**	**OC relevance**	**References**
**CYTOKINE RECEPTORS**			
IL-2R	IL-2	NK cells isolated from OC patient ascitic fluid demonstrated reduced proliferation in response to interleukin-2 (IL-2)	(21)
IL-10R	IL-10	In OC patient ascitic fluid increased IL-10 expression relates to advanced stages (III/IV)	(22)
IL-12R	IL-12	Human PBMCs, isolated from OC patients, stimulated by IL-12 demonstrate enhanced activation and proliferation of functional NK cells	(23)
IL-15R	IL-15	Increased levels in OC patient ascitic fluid were associated with increased NK cell cytotoxicity	(24)
IL-21R	IL-21	Mice treated with IL-21 demonstrated delayed tumor appearance and reduced OC tumor size	(25, 26)
TGF-βR	TGF-β	Increased TGF-β expression in OC tumors has been associated with progression and metastasis	(27–30)
**ACTIVATING RECEPTORS**			
2B4 (CD244)	CD48	Downregulation of 2B4 and hyporesponsiveness of 2B4+ NK cells to MHC class I -negative targets in OC patient ascitic fluid	(31–34)
CD16 (FcRγIII)	Fc portion of antibodies	Decreased expression has been identified in NK cells isolated from OC patient ascitic fluid	(21)
CD69	Undefined	Increased expression has been identified in NK cells isolated from OC patient ascitic fluid	(24)
DNAM1 (CD226)	CD155, CD112	Decreased DNAM1 expression and hypo-responsiveness of DNAM1+ NK cells to MHC class I – negative targets in NK cells isolated from OC patient ascitic fluid	(24, 31–33, 35)
NKG2D	NKG2D ligands – various, including MIC-A/B, ULBP1-6	NKG2D was downregulated on NK cells isolated from OC patient ascitic fluid	(24, 35, 36)
NKp30 (CD337)	Various, including B7-H6, CMV pp65 tegument protein, BAG6, heparan sulfate	Decreased NKp30 expression on NK cells isolated from OC patient ascitic fluid	(35, 37)
NKp44 (CD336)	Various, including proliferating cell nuclear antigen (PCNA), platelet-derived growth factor (PDGF), mixed-lineage leukemia-5 (MLL-5), viral hemagglutinins	Decreased NKp44 expression on NK cells isolated from OC patient ascitic fluid	(24, 33, 38)
NKp46 (CD335)	Various, including complement factor P, heparin sulfate, viral hemagglutinins	Decreased NKp46 expression on NK cells isolated from OC patient ascitic fluid	(24, 35)
TRAIL	TRAIL-R	TRAIL-R downregulated on OC cells isolated from OC patient tumors	(39)
**INHIBITORY RECEPTORS**			
KIR2DL1 (CD158a)	MHC-C2 group ligands	Decreased expression on NK cells isolated from OC patient ascitic fluid	(33)
KIR2DL2 (CD158b)	MHC-C1 group ligands (major); some binding to MHC-C2 group ligands	Decreased expression on NK cells isolated from OC patient ascitic fluid	(33)
KIR2DL3 (CD158b)	MHC-C2 group ligands	Decreased expression on NK cells isolated from OC patient ascitic fluid	(33)
KIR3DL1 (CD158e)	MHC-B alleles with the Bw4 motif	Decreased expression on NK cells isolated from OC patient ascitic fluid	(33)
PD-1	PD-L1	PD-1 overexpression on NK cells isolated from OC patient ascitic fluid	(40)

NK cell responsiveness varies within and between individuals, via a process called “education,” “licensing,” or “arming.” The killer immunoglobulin-like receptors (KIR) interact with conserved epitopes on HLA (41). KIR and HLA genes are both highly polymorphic and polygenic and their loci segregate independently (42). Thus, the availability of binding pairs differs between people, with consequences on NK cell reactive potential (42). NK cells expressing KIR that can bind to “self” HLA (licensed) are highly responsive to targets lacking “self” HLA—a process termed “missing self” recognition. NK cells that do not express self-HLA specific receptors are “unlicensed” and require potent stimulation for reactivity. Unlicensed NK cells are especially protective against HLA-expressing tumors because they are refractory to the inhibitory signals sent by HLA (43, 44). Thus, education potentiates a spectrum of NK cell reactivity, establishing, at the repertoire level, an array of functions to target HLA-sufficient and HLA-deficient target cells (19, 20).

NK cells are capable of memory or adaptive functions, whereby previous sensitization leads to expansion and retention of a population of NK cells that respond more rapidly and robustly to secondary challenges with the same virus or hapten (45, 46). These “adaptive” NK cells have undergone epigenetic alterations leading to a distinct phenotype and enhanced function (47–49). While additional research is required to fully characterize this cell population, unbound inhibitory receptors, NKp44, NKp46, NKG2C, and CD57 receptors are consistently overexpressed (47–50). Noteworthy, these same adaptive features can be achieved by cytokine cocktail stimulation in the absence of a specific antigen; cytokine-induced adaptive cells exhibit increased expression of activating receptors including NKp30, NKG2D, NKp44, NKp46, and TRAIL (51). Thus, while a memory response may be generated toward a specific pathogen, it may be the environment or steric changes in the HLA-peptide complex that drive NK cell adaptive functions (45). For cancer immunotherapy, this is noteworthy as it opens the possibility to train highly effective NK cells without strict requirements for antigen restriction.

## The Tumor Microenvironment and Immunosuppression in Ovarian Cancer

**Figure 1:**
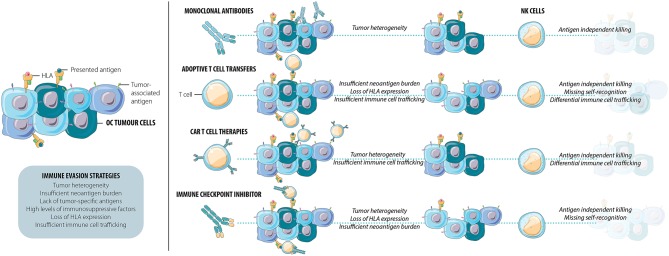
Current immunotherapies frequently result in the persistence of a resistant cell population. This immune evasion of OC tumor cells can be facilitated by tumor heterogeneity, insufficient neoantigen burden, lack of tumor-specific antigens, high levels of immunosuppressive factors, loss of HLA, and/or insufficient immune cell trafficking. NK cells exhibit multiple functions that combat immune escape and tumor relapse: they kill targets and elicit inflammation through both antigen-specific and antigen-independent pathways and detect loss of HLA as a signal for activation. As efficient mediators of tumor immune surveillance and control, NK cells may be able to kill the cells many current immunotherapies leave behind.

The tumor microenvironment (TME) includes the tumor, stroma, and local immune cells. The TME is dynamic and can promote or suppress tumor invasion and metastasis. The immune composition in the TME is now known to be an important predictor of response to therapy in a variety of solid tumor types, including OC. Several factors, processes, and cellular subsets contribute to the development of the TME including innate and adaptive immune cells, intercellular signaling, and tumor intrinsic factors such as gene mutational burden (Figure 1) (52).

**Figure 2:**
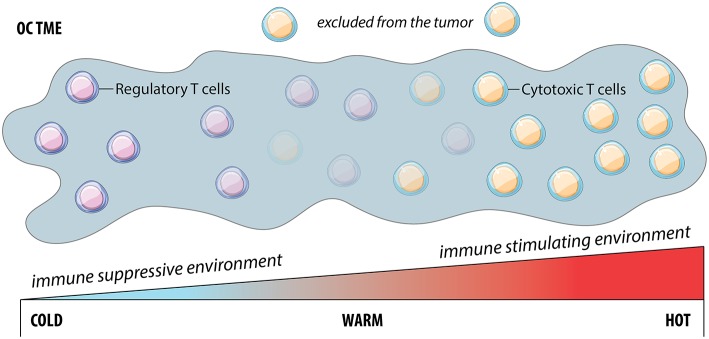
The cold, warm or hot tumor microenvironment is a continuum. Cold tumors are characterized by the lack of cytotoxic T cells and are typically associated with an immune suppressive environment. Conversely, hot tumors are well infiltrated by cytotoxic T cells and are associated with an immune stimulating environment and better prognosis than the prior. However, the characterization of tumors is not dichotomous, but rather exists as a sliding scale of cytotoxic T cell infiltration with “warm” tumors representing a situation where although T cells exist, they are excluded and therefore ineffective at producing an efficient anti-tumor response.

Good prognosis for OC and its treatment are correlated with immune cell infiltration in the TME, and the majority of studies focused on infiltrating T cells (53) (Figure 2). These so-called “hot” tumors represent two scenarios: (1) immune inflamed tumors, where T cells can directly contact malignant cells; and (2) immune excluded tumors, where T cells are restricted to the stroma surrounding malignant cells, with limited access for ligation and killing. In contrast, “cold” or “non-inflamed” tumors lack T cell infiltration. Reproducibly, patients whose OC tumors are infiltrated by T cells respond better to therapy and have better prognosis (54, 55): 55% of patients with T cell infiltration reached a 5-year survival of 38% compared to just 4.5% in patients without T cell infiltration (54).

Tumor transcriptome analysis has revealed correlations of OC sub-classifications with T cell infiltration, neoantigen burden and patient prognosis, termed C1, C2, C4, and C5 (8). Among them, the C2 “immunoreactive” subtype is well-infiltrated and associated with the best prognosis (8). Intermediate T cell infiltration and prognoses are associated with the C1 “mesenchymal” and C4 “differentiated” subtypes, and the C5 “proliferative” has the lowest T cell infiltration and conveys the poorest prognosis (8). Despite the mutational burden found in many OC—a feature that can predict priming and infiltration of lymphocytes—they remain immunologically “cold,” and infiltrating T cells are not universally reactive against tumor antigens (56, 57). This highlights an urgent need to better understand how other cellular and acellular players contribute to tumor growth and treatment success.

**Figure 3:**
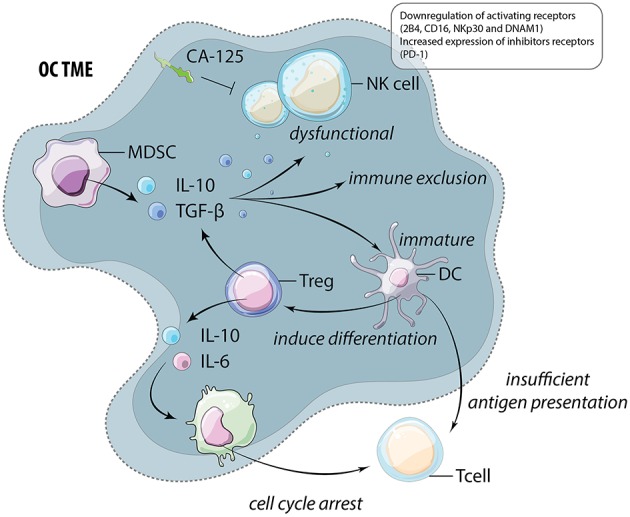
The immunosuppressive TME of OC. Myeloid derived suppressor cells (MDSC) and regulatory T cells (Treg) release high levels of immunosuppressive cytokines including IL-10 and TGF-β. These cytokines function to alter and suppress the function of multiple cells within the TME including natural killer (NK) cells, dendritic cells (DC), and macrophages (M). These cells frequently feedback onto lymphocytes by inducing further Treg differentiation and preventing anti-tumor cytotoxic T cell function through insufficient antigen presentation and the induction of cell cycle arrest.

An additional barrier to immunotherapy and effective anti-cancer reactivity is the immunosuppressive nature of the TME, which advances with OC progression (58). Dendritic cells (DCs) are required for the activation of anti-tumor T cells but also have the ability to release immunosuppressive cytokines. In OC, DCs are dysfunctional and immature due to high levels of VEGF and IL-10 in the TME (59–61). These DCs are not only unable to activate cytotoxic T cells, but also function to induce regulatory T cell (Treg) differentiation, further promoting immune suppression (62). Moreover, patient ascitic fluid contains high levels of the Treg recruiting chemokine, CCL22 (63). Unsurprisingly, Treg accumulate in the ascites isolated from late stage OC patients (64, 65). Treg release IL-6 and IL-10 which induces expression of B7-H4 on macrophages and subsequently leads to cytotoxic T cell cycle arrest (65). Discussed in the following section, these impacts extend beyond suppression of T cell immune responses and likely also interfere with productive, anti-tumor NK cell function (Figure 3).

### NK Cells in the OC Tumor Microenvironment

The important contributions of NK cells to cancer control were identified through mouse models deficient in NK cells or key NK cell activating receptors (66, 67). The protective effect of NK cells was further supported by studies in humans that correlated poor NK cell function to cancer susceptibility, progression and metastases in a variety of both solid and hematologic cancers (68, 69). In OC, the prognostic value of infiltrating NK cells has been debated—NK cells and the NK cell-like population, innate-like lymphocytes (ILCs), have been associated with both tumor progression and control (70–72). NK cells co-infiltrate with cytotoxic T cells and are strongly associated with patient survival (71, 73). One study stratified OC patients into three subgroups based on infiltration of T cells and other lymphocytes, primarily NK cells, and reported differential 5-year survival rates: T + NK cells (90%), NK cells only (63%) and neither T nor NK cell infiltration (0%) (70). Even when they are present in OC, NK cells are dysfunctional: they exhibit reduced proliferation, decreased cytolytic function and decreased inflammatory cytokine production compared to the same patient's peripheral blood NK cells (74, 75) (Summarized in Table 1). Hence, a better understanding of how to enumerate, assess and control NK cells will be required to maximize their infiltration, function and reactive potential in the TME.

Alterations in NK cell phenotype and function occur as a result of the products of a growing ovarian tumor, related ascites, and a variety of immunosuppressive cytokines produced by myeloid derived suppressor cells (MDSC) and Treg (76). For example, macrophage migration inhibitory factor (MIF) overexpression has been reported in OC and correlates with tumor progression (77). MIF downregulates transcription of the NK activating receptor NKG2D (37, 77, 78). MIF is also associated with increased expression of the inhibitory checkpoint, B7-H6, which is associated with overall poorer prognosis in OC (34, 79). Similarly, TGF-β overexpression can suppress CD16-triggered NK cell IFN-γ production (80) and, together with IL-10, has been shown to decrease the inflammatory cytokine production and cytotoxicity of various effector cells including NK cells (81–84). These alterations include a downregulation of activating receptors 2B4, CD16, NKp30, DNAM1, and an upregulation of the inhibitory checkpoint receptor PD-1 (21, 31, 32, 37, 40). Finally, despite upregulation of the early activation marker, CD69, NK cells expressing it remain poorly cytolytic (31). In addition to cytokine mediated suppression, CA-125, an antigen overexpressed in 80% of OC, can directly protect tumor cells from NK cell-mediated cytotoxicity by preventing the formation of an immune synapse, regardless of the repertoire of receptors expressed on the NK cells (79, 85).

Taken together, the available research indicates that NK cells interact dynamically with the OC TME and are highly sensitive to the immunoregulation it drives. Infiltration of NK cells into a tumor may only partially predict outcomes of OC. Better understanding the mechanisms through which the TME contributes to immune exclusion and dysregulation will be required for precise tumor control and to maximize NK cell reactivity.

## OC Immunotherapy: A Focus on the Roles and Potential of NK Cells

Successful immunotherapy requires restoration of immunity in the immunosuppressive TME and complete targeting of a heterogeneous tumor. Anti-tumor immunity can be driven by *ex vivo* re-stimulation of lymphocytes, engineering cells for direct targeting of specific tumor-associated antigens or turning off immune suppression (86–90). Antibody-based therapies can redirect immune cells by blocking their function, or for antibody-dependent cell-mediated cytotoxicity (ADCC), a process for which NK cells are major effectors. While the majority of immunotherapeutic approaches have been developed with a goal of supporting or reinvigorating antigen-specific anticancer activity, they can also support the function of NK cells, whose functional features can complement and extend the breadth of OC immunotherapy (Figure 1). In the following sections, we highlight current approaches to cancer immunotherapy, their potential interactions with NK cells and the opportunities to maximize anti-tumor immunity by recruiting NK cells.

### Cytokine-Based Immunomodulation

Recognizing that immunosuppression is a major hindrance for lymphocytes to proceed in anti-cancer activity, approaches with cytokines to induce local and/or systemic inflammation have been tested. A strategy to elicit and improve immune cell activation in humans was first attempted using a variety of activating cytokines including IL-2, IL-12, IL-15, IFN-α, and IFN-γ (91).

IL-2 was one of the earliest cytokines tested for improving anti-tumor immunity. Although early clinical trials were limited by toxicity and activation of Treg, they provided an important proof of concept that stimulating T and NK cells can impact tumor progression. Since then, research has focused on strategies to improve IL-2 safety including low-dose IL-2. In patients with platinum-sensitive advanced OC, low-dose IL-2 in combination with 13-cis-retinoic acid improved clinical outcomes and increased lymphocyte and NK cell counts (92). As low-dose IL-2 can activate Treg, current efforts are testing constructs that selectively bind to NK cells to support anti-tumor immunity without driving Treg proliferation (93, 94).

Similar disappointing and toxicity-related issues were reported in many trials of activating cytokines. Research resulting in the development of analogs and oncolytic strategies for local delivery may provide the required specificity to bring cytokines safely into clinical use. IL-15 is similar to IL-2 but more specific in that it binds cytotoxic T cells and non-terminally differentiated NK cells to enhance cell cytotoxicity and proliferation. Further, the toxicity of IL-15 is less than that of IL-2, but the concentrations of IL-15 required to drive efficient anti-tumor function remain toxic. Ongoing efforts involve IL-15 “superagonists,” which deliver the IL-15 signal in complex with the IL-15 receptor alpha subunit or its biologically-relevant fragments, and/or fused in dimers with an IgG1Fc molecule to stabilize the complex. In each instance, these superagonists more closely replicate the biologically-potent delivery of IL-15, exhibit longer *in vivo* half-lives, and drive lymphocytes (including NK cells) for anti-cancer activity without marked toxicity (95).

ALT-803 is an IL-15 superagonist that potently enhances NK functionality *in vitro* and *in vivo* against OC cell lines (96). After ALT-803 treatment, NK cells isolated from OC patient ascitic fluid exhibited greater degranulation (CD107a) and IFN-γ production (24). Several clinical trials are ongoing evaluating the efficacy of ALT-803 and other IL-15-based therapies, alone and in combination with other immunotherapies including three for patients with OC: NCT03054909, NCT03197584, and NCT03127098 (97). It is expected that the addition of IL-15 and its related superagonists will support NK cell proliferation and development. Metrics to understand NK cell recruitment to the OC TME, persistence and NK cell reactivity (i.e., with *ex vivo* restimulation) will enlighten subsequent clinical trials by indicating how NK cell reactivity can be improved further. Furthermore, studies to understand whether the cytokine milieu varies with defined OC subtypes might help to predict how NK cells will be recruited and effective in patients with OC.

### Checkpoint Blockage and Antigen Insufficiency

A high mutational burden creates a challenge for antigen-targeted immunotherapies, but it creates an opportunity for immune-mediated OC recognition. Tumors with high mutational burdens may have increased neoantigen levels, against which antigen-specific T cells may be activated. Many studies have predicted that resistant tumors may lack the neoantigen burden required to mount an effective T cell response (98). One recent investigation profiling tumor and T-cells isolated from the ascites of three OC patients identified that while a high mutational burden was indeed present, only 1.3% of these mutations were recognized by tumor-associated T cells (99). As expected, the presence of neoantigen-reactive T cells (rather than T cells with non-specific reactivity) is predictive of improved prognosis in OC (99, 100). This highlights the importance of the immunogenicity of antigens and an adequate repertoire of tumor-reactive T cells, rather than the overall tumor mutational burden in predicting OC outcomes (101). However, the immune-suppressive TME can still interfere with T cell-mediated tumor recognition.

High mutational burdens have been associated with improved responses to immune checkpoint therapies in both melanoma and non-small cell lung cancers (102, 103). Unexpectedly, despite a significant mutational load being present in pre-treatment surgical samples of OC patients, overall response rates to anti-PD1 treatment in clinical trials have been largely disappointing, ranging from 11–24% (104, 105). These clinical trials were mainly conducted in OC patients that progressed despite conventional treatments—these tumors likely had established immune evasion strategies; earlier intervention with immunotherapy may have achieved better outcomes. Regardless, these clinical trials highlight that a subset of OC patients can respond to anti-PD1 treatment, but the majority have innate or acquired resistance or lack T cells with appropriate anti-tumor reactivity (106).

Various changes in the TME have been identified that may be associated with anti-PD1 resistance including genomic mutations and downregulation of HLA and its associated processing and presentation pathway components (106, 107). These alterations have been reported in both melanoma and lung tumors treated with anti-PD1 (106, 107). Specifically, the acquired genomic mutations provided protection against T-cell mediated killing via loss of IFN-γ or HLA class I components (108, 109). It is not yet known whether anti-PD1 therapy drives similar genetic alterations in OC, but in recurrent OC the expression of HLA genes was negatively correlated with expression of PD-L1, suggesting two mutually exclusive pathways to immune evasion (110). This loss of HLA class I expression, together with insufficient T cell-mediated recognition of tumor antigens, may contribute to the insufficiency of anti-PD1 for complete OC clearance. Strategies to augment NK cell function, particularly those NK cell populations that recognize cells with DNA damage and loss of components of the HLA processing and presentation pathway (i.e., the “licensed” NK cell population), may improve OC treatment and the outcome of anti-PD1 therapy, and complement existing strategies aimed at maximizing T cell-mediated OC control. Likewise, phenotyping of tumor-infiltrating NK cells or the tumors themselves for expression of checkpoints and HLA expression may assist in predicting how NK cells may be functional or inhibited against tumor killing.

Checkpoint inhibitors interfere with inhibitory signaling that prevents anti-tumor reactivity; their application enables lymphocytes to proceed in anticancer cytotoxicity. Although anti-PD1 therapies were designed to rescue T cells from immunosuppression, PD-1 has been found to be expressed on NK cells isolated from OC and other tumor types (40, 111, 112). PD-1 expression is not universal on NK cells however, with studies reporting highly variable PD-1 expression peripheral NK cells in healthy donors, from 0 to 50% (40, 113). In patients with OC however, PD-1 expression on peripheral blood NK cells is increased suggesting that the presence of a tumor could induce its expression (40).

Despite inconsistent expression on NK cells, anti-PD1 therapies have demonstrated the potential to simultaneously support T and NK cell responses in the TME, suggesting that PD-1 blockade could indirectly influence NK cell function, or that PD-1 expression could be dynamic on NK cells in response to the TME. A recent study investigated the therapeutic effect of anti-PD1 therapy on NK cells using several mouse cancer models and concluded that NK cells were crucial to anti-tumor responses (114). Additionally, OC xenograft studies have demonstrated that both NK cell persistence and cytotoxicity can be improved with PD-L1 blockade (115). Hence, NK cells may be contributing to the outcomes of checkpoint inhibition therapy, even if they have not been expressly studied for this purpose. Recognizing this potentially important feature, clinical trials have also begun to investigate the impact of anti-PD-1 treatment on preventing or reversing NK cell exhaustion in the TME (NCT03241927), but this is not yet standard in clinical trials. Given that NK cells may contribute to the anti-tumor immunity driven by checkpoint inhibition therapies, metrics to assess their prevalence and function may help to illuminate the larger picture of anti-OC immunity.

Some studies have identified an immunoregulatory population of NK cells that exhibit the PD-1 ligand PD-L1 in patients with cancer, but its function has not been determined (116). In antigen presenting cells *cis* expression of PD-L1 and PD-1 permits regulation of PD-1 signaling (117); whether this also occurs in NK cells is unknown. An alternative mechanism, demonstrated in a mouse model, involves PD-L1-mediated editing of dendritic cells, limiting the extent of their interactions with T cells and development of productive anti-tumor T cell response (118). With this in mind, expression of PD-L1 (and its control by anti-PDL1 antibodies) may have significant impacts on direct NK cell function and its interactions with neighboring cells; this warrants further investigation.

In addition to PD-1, other immune checkpoints, including KIR, NKG2A, and TIGIT are being explored as targets for immunotherapy (119, 120). Like PD-1, TIGIT is expressed on both T and NK cells and suppresses anti-tumor effector function in both (120). While its mechanism of action in T cells was recognized, it was only recently identified that they also prevent or reverse NK cell exhaustion (120). TIGIT blockade improved prognosis in murine T and B-deficient, NK-sufficient xenograft models against several human tumor cell lines including colon, breast, melanoma, and fibrosarcoma (120). Anti-TIGIT antibodies are now undergoing clinical trials and just one includes OC (NCT036286770). This trial does not plan measurements of NK cell phenotype or function; future studies should include this as an important outcome measure.

Like TIGIT and PD-1, NKG2A, and the inhibitory subset of KIRs prevent excessive inflammation but may interfere with productive anti-cancer activity. For NK cells, NKG2A and KIR convey an important signal of “self” upon binding with HLA class I. The importance of preventing NK cell inhibition is clear in patients undergoing hematopoietic cell transplantation for acute myelogenous leukemia, where “uninhibitable” populations of NK cells predict for less relapse and greater overall survival (19, 121–123). These observations inspired the creation of antibodies against KIR and NKG2A, which are now being tested against other hematologic and solid malignancies, but not yet in OC.

HLA-E, the ligand for NKG2A, is a non-classical and ubiquitous HLA molecule that has been found at high expression levels in OC tumors (124). As HLA-E overexpression is negatively correlated with survival and exhibited in the majority of tumor types, blocking this NKG2A/HLA-E inhibition could enhance immunotherapy across an array of tumors, including OC (125, 126). In tumors exhibiting high-density HLA-E expression, infiltrating CD8+T and NK cells exhibit high NKG2A and PD-1, suggesting adoption of a phenotype highly sensitive to inhibition in the TME (126). Although OC-infiltrating NK cells' NKG2A expression has not been expressly measured, the abundance of HLA-E on OC tumors would suggest that preventing inhibition of NK cells via NKG2A may enable strong anti-tumor reactivity.

Experimentally, NKG2A surface expression has been eliminated on NK cells by blocking its export from the endoplasmic reticulum using a protein expression blocker construct (126). Primary human NK cells engineered in this way lacked NKG2A surface expression and more efficiently controlled growth of human tumor xenografts in mice (126). Toward a similar goal, a monoclonal antibody, anti-NKG2A (monalizumab) has been delivered for direct delivery to patients. In addition to boosting NK cell cytotoxicity against targets expressing HLA-E, anti-NKG2A has also been demonstrated to augment the function of T cells expressing NKG2A, providing an opportunity to activate both NK and T cells; both were shown to contribute to control of tumor xenografts in mice (127). Inclusion of NKG2A and HLA-E measurements in OC tumors from patients could help to ascertain whether a patient might benefit from anti-NKG2A therapy.

The anti-KIR antibody IPH2101 (lirilumab) aims to interfere with inhibition via the KIR2DL1/2/3 receptors. *In vitro*, lirilumab functions to enhance killing of tumor cells by NK cells (128). Unfortunately, efficacy for this monoclonal antibody has been poor in clinical trials for patients with hematologic malignancies (129), a finding that corresponds with decreased surface density of KIR molecules and “detuning” or diminishment of missing self-responsiveness (130). IPH2101 has not yet been tested against OC, but the available information suggests that further improvements, such as interrupting inhibitory signaling without altering receptor expression, preventing the loss of cell surface KIR through the trogocytosis prompted by IPH2101, or stratifying patients based on particular KIR haplotypes or KIR allotypes likely to be sensitive to lirilumab, will be necessary to gain efficacy against OC.

By combining checkpoint inhibitors, it may be possible to augment the NK cell anti-tumor response—by relieving two or more inhibitory signals, or by rebalancing immunity toward activation by blocking inhibition while triggering activation. These approaches could lower the threshold for NK cell activation and provide a failsafe to target a tumor that evolves away from dependence on PD-L1 and/or HLA expression. Unlike T cells, where TIGIT and PD-1 are often co-expressed, TIGIT and PD-1 expression was nearly mutually exclusive on NK cells, suggesting that checkpoint blockade for both molecules simultaneously may permit rescue of a larger population of NK cells from inhibition and exhaustion (120). Simultaneous blockade of NKG2A and triggering of ADCC using the anti-EGFR antibody cetuximab enhanced *in vivo* control of tumor xenografts more efficiently than either antibody alone (127). In a phase II trial (NCT02643550), monalizumab was given to patients with squamous cell carcinoma of the head and neck after chemotherapy and alongside standard-of-care treatment with cetuximab (127). This combination was deemed safe, with interim results indicating an improvement from the addition of monalizumab compared with historical control subjects treated with cetuxumab only, but further studies will be required to formally draw this conclusion.

Clinical trials are now testing combinations of anti-PD1 with novel checkpoint inhibitor therapies including anti-KIR2D antibody (lirilumab) (NCT01714739), anti-NKG2A (monalizumab) (NCT02671435, NCT03822351, NCT02557516, NCT03794544, NCT03833440), and anti-TIGIT (MTIG7192A) (NCT02794571, NCT03119428, NCT03563716, NCT03628677) (127). Theoretically, further strategies to appraise the tumor's expression of PD-L1 (which is the current standard of care for checkpoint therapies in other cancers), HLA and TIGIT ligands may inform the rational combination of checkpoint inhibitors to maximize NK cell function.

### Adoptive and Adaptive NK Cell Therapies

Recognizing the potential for NK cells to participate in immune-mediated cancer control, a thrust in NK cell-based cancer therapies is adoptive transfer of NK cells (131, 132). Since NK cells do not require HLA matching to a specific patient, it is feasible and safe to transfer cells across allogeneic barriers. This opens the possibility of transferring NK cell lines (i.e., NK-92) or *ex vivo*-expanded NK cells from third-party donors (25). Efforts are underway to create cell lines—including those based on NK-92 cells—which may enable direct targeting of OC based on defined criteria (133, 134). In addition, clinical protocols are in place for virtually unlimited expansion of primary NK cells for adoptive transfer. Together, these efforts open the possibility of off-the-shelf NK immunotherapy. A complete summary of all clinical trials employing NK and NK-related cells for treatment of OC is shown in Table 2.

**Table 2:** Current NK cell-based adoptive cell immunotherapies under clinical trial for the treatment of ovarian cancer.

**NK cell intervention**	**Phase, date, (Status)**	**Study population (*n*)**	**Primary outcomes**	**Results**	**Reference/Clinical trial identifier**
Allogeneic NK cells (with IL-2)	Phase II, 2008–2010 (Terminated due to toxicity)	Ovarian cancer, fallopian tube cancer, peritoneal cavity cancer (12)	To evaluate the *in vivo* expansion of an infused allogeneic natural killer (NK) cell product	PR (3), SD (8), PD (1)	NCT00652899
Allogeneic NK cells (with IL-2)	Phase II, 2010–2014 (Completed)	Ovarian cancer, fallopian tube cancer, primary peritoneal cancer, breast cancer (13)	Response Rate by RECIST [Time Frame: Month 3]	N/A	NCT01105650
Cord Blood Cytokine Induced Killer Cells	Phase I, 2012–2014 (Completed)	Ovarian (4), colon (4), rectal (5), hepatocellular (2), gastric (1), pancreatic (1), lung (1), esophagus (1)	Response Rate by RECIST	CR (1, HCC, 1 esophageal), PR (2 ovarian), PD (1 HCC), SD (10, averaging 11.4 months)	(135)
Radiofrequency ablation and Cytokine-induced Killer Cells	Phase II, 2015–2016 (Active, not recruiting)	Ovarian carcinoma (50)	Recurrence-free survival [Time Frame: 1 year]	N/A	NCT02487693
NK cells with cryosurgery	Phase I/II, 2016 (Recruiting)	Recurrent ovarian cancer	Response Rate by RECIST	N/A	NCT02849353
FATE-NK 100 (CMV+ donor NK cells with IL-2)	Phase I, 2017–2019 (Recruiting)	Epithelial ovarian cancer, Fallopian tube cancer, Primary peritoneal cancer (*estimated* 16)	Maximum Tolerated Dose of FATE-NK100 [Time Frame: 1 Year]	N/A	NCT03213964
Primary NK cells	Phase I/II, 2018 (Recruiting)	Lung cancer, breast cancer, colon cancer, pancreatic cancer, ovarian cancer (*200*)	Incidence of toxicity induced by NK infusion [Time Frame: 6 months]	N/A	NCT03634501
NKG2D-Ligand Targeted CAR-NK	Phase I, 2018 (Recruiting)	Solid tumors (*estimated* 30)	Number of Adverse Events [Time Frame: from day 0 to month 4]	N/A	NCT03415100
6B11-OCIK	Phase I, 2018 (Not yet recruiting)	Recurrent platinum-resistant ovarian cancer (*estimated* 10)	Progress-free survival [Time Frame: 3 years]	N/A	NCT03542669
Allogeneic NK cells	Phase I, 2018 (Not yet recruiting)	Recurrent ovarian cancer, recurrent fallopian tube cancer, recurrent primary peritoneal cancer (*estimated* 12)	Incidence of treatment emergent adverse events [Time Frame: 6 months]	N/A	NCT03539406
Anti-Mesothelin CAR-NK	Phase I, 2018 (Not yet recruiting)	Epithelial ovarian cancer (*estimated* 30)	Occurrence of treatment related adverse events as assessed by CTCAE v4.0 [Time Frame: Day 3-Year 2 after injection]	N/A	NCT03692637

*SD, stable disease; PR, partial response; CR, complete response; PD, progressive disease*.

Early attempts at inducing NK cells for anti-cancer function included priming of lymphokine activated killer (LAK) and cytokine induced killer (CIK) cells. LAK and CIK cells originate from naïve lymphocytes which are “activated” or “induced” by IL-2 alone (LAK) or following IFN-γ stimulation *ex vivo* (CIK) (136, 137). In clinical trials, LAK cells exhibited limited clinical response and high rates of peritoneal fibrosis (138–140). The addition of IFN-γ stimulation to LAK cells to create CIK cells substantially improved both proliferation and cytotoxicity. CIK cells are characterized by the expression of a CD3+CD56+ cell phenotype and functional properties of NK cells (141). CIK cells were used in a recent phase III study of adoptive transfer following primary debulking and carboplatin/paclitaxel chemotherapy of OC (141, 142). Results from this clinical trial were positive with progression free survival improving from 22.2 months in the control group to 37.7 months among CIK-treated patients (142).

A completed early phase II study used allogeneic, haploidentical donor NK cells in combination with high-intensity chemotherapy to treat patients with recurrent OC (143). NK cell effects were difficult to differentiate from those of the chemotherapy and limited efficacy was attributed by the authors to a significantly increased number of Treg. Overcoming immunosuppression, including that driven by Treg, will indeed be important to ensuring the efficacy of NK cell-based immunotherapy. One strategy is to combine haploidentical donor NK cells with cytokines, with the goal of reversing the suppressive immune TME, enabling NK cell anticancer reactivity to proceed. Supporting this, the authors of the aforementioned phase II study have recently reported the ability to stimulate NK cells to overcome the soluble immunosuppressive environment from OC patient ascites *in vitro* with a combination of stimulatory IL-12, IL-15, and IL-18 (144). Indeed, this cytokine cocktail has been known to induce an “adaptive” population of highly-functional NK cells (145).

“Adaptive” NK cell population, first identified following cytomegalovirus infection but now known to be long-lived effector cells with potent cytotoxic ability (47). A recent study converted NK cells from a patient with OC to a cytotoxic CD56superbrightCD16+ subset that upon autologous transfer efficiently controlled growth of an autologous OC xenograft in a mouse (146). These adaptive NK cells are refractory to inhibition by Treg, implying a mechanism for their enhanced function (147). In a current Phase I clinical trial, FATE-NK100 cells, which are primary NK cells isolated from haploidentical cytomegalovirus-seropositive donors, are being transferred to patients with OC (NCT03213964).

Identifying the ideal source of NK cells for adoptive immunotherapy is a field of active study. Compared with mature and *in vitro* differentiated NK cells, an alternate approach is to transfer NK cells derived from umbilical cord blood (UCB) stem cells (NCT03539406). UCB contains a high proportion of immunologically-naïve NK cells that can be easily recovered and exhibit functions similar to peripheral blood NK cells. They produce similar amounts of IFN-γ and TNF (148–150). Research has also highlighted some potential weaknesses of UCB-derived NK cells: the relative immaturity of this NK cell population is associated with lower cytotoxicity, lower expression of perforin, granzyme B and KIR, and higher expression of NKG2A (150, 151). The processes for NK cell differentiation, the relative immaturity of NK cells from UCB and how they can be further differentiated or potentiated using cytokine cocktails or stimulation remain to be studied.

While selection of where to source NK cells from continues to elicit debate, once transferred into a patient, persistence, expansion, and homing/trafficking of the NK cells has proven an additional challenge. Unsurprisingly these factors have been identified as key to anti-tumor efficacy (143, 152, 153). Several factors including modifying cryopreservation and cytokine stimulation techniques can have profound impacts on homing, persistence and expansion of NK cells in *in vivo* (154). Indeed, a variety of strategies are being tested to improve NK cell potency *in vivo*. Interestingly, a recent study describes the use of a NK-cell-recruiting protein conjugated antibody that is cleaved into CXCL16 upon interaction with the tumor surface. This created a chemokine gradient resulting in increased NK cell tumor infiltration in a mouse model of pancreatic cancer (155). Similar strategies could be employed for other solid tumors including OC. Likewise, inclusion of extensive immune phenotyping, including that which would identify the type(s) and relative differentiation of NK cells invading tumors in patients, with stratifications based on outcomes and tumor subtypes, might better inform precise application of adoptive NK cell therapies.

### Antigen Targeting With Antibodies and CAR-NK Cells

T cells engineered to express a chimeric antigen receptor (CAR)-T cells targeting CD19, a tumor antigen present on B-cell malignancies, provided the groundwork, and rationale for development of T cells engineered to target a specific antigen. Likewise, antibodies against CD20 and the HER2 receptor on lymphoma and breast cancer, respectively, provided the first proof-of-principle that tumors can be tagged for recognition and elimination by the immune system. Noteworthy, many of these antibody therapies rely on NK cells to mediate ADCC, and their efficacy has been inversely correlated to the extent of inhibitory KIR expression on NK cells (43, 156, 157). The list of targetable antigens in OC has been growing, and currently includes CA-125, FOLR1, EPCAM, MUC-1, and NY-ESO-1 (158, 159). Unfortunately, immunotherapies targeting these specific antigens have been largely ineffective. Currently, two CAR-T cell therapies have been approved by the FDA (160); neither is applicable to OC. For patients with OC, CAR-T cells against CA-125 are being developed and have shown promise against human xenograft models and plans to evaluate their safety in in-human phase I clinical trials have been reported (161, 162).

There are limitations to the universal use of CAR-T cells, which may be directly addressed by NK cells: CAR-T cells take weeks to produce, are difficult to generate as autologous, and expensive, making them impractical for patients requiring quick treatment for aggressive tumors or standard of care therapy (163). Moreover, allogeneic CAR-T cells pose the risk of graft-vs.-host disease (GvHD), even when HLA-matched, due to minor mismatches (164).

Since NK cells can be delivered from allogeneic sources, they are readily available and relatively more cost-effective. Importantly, NK cells are not associated with GvHD, and therefore CAR-NK cells may be a safer alternative to CAR-T cells for engineered cell therapy. Preliminary data supports the safety and efficacy of CAR-NK cell therapy, which may be attributed to the relatively short lifespan of NK cells, which lowers the risk of long-term autoimmunity and toxicity (165). Moreover, the pre-existing tolerance conveyed by germline-encoded NK cell inhibitory receptors (i.e., KIR, NKG2A) may restrict their reactivity to the tumor and damaged cells, making them less likely to convey off-target and toxic effects than CAR-T cells. Two CAR-NK cells currently under development include CARs against CD24 and mesothelin.

CD24, a cancer stem cell marker, is rarely expressed in non-hematologic healthy tissue, and associated with poor clinical outcome in OC patients (166, 167). Recently published work tested a genetically engineered CAR-NK92 against CD24, and demonstrated its to ability kill OC cells *in vitro*, in addition to producing high levels of IFN-γ upon co-culture with CD24 expressing OC cell lines (168). Researchers indicate that future *in vivo* experiments will be conducted to further evaluate efficacy.

One of the most developed CAR-NK cells is engineered against mesothelin, the receptor for CA-125. CA-125 is overexpressed in ~80% of patients and elicits an effective T cell response *in vitro* (169). A recent study evaluating various CAR constructs against mesothelin demonstrated their cytotoxic potential *in vitro* and further proved to be less toxic than their CAR-T cell counterparts *in vivo* while retaining similar anti-tumor effects (170). Based on early success *in vivo*, there are plans to evaluate mesothelin targeting CAR-NK cells in a phase I clinical trial (NCT03692637).

Despite its high expression on OC tumors, antibodies against CA-125 have not proven efficacious in OC patients (171), suggesting that exclusively targeting CA-125 is insufficient to target the heterogeneity associated with primary OC. “Antigen escape” in which the target antigen is lost through downregulation, acquired gene mutation or outgrowth of tumor subpopulations, occurs as a result of the selective pressure applied by antigen-targeting therapies like CAR-T cells and antibodies. This likely underlies the incomplete tumor control by existing antigen-specific immunotherapies and is facilitated by the tumor heterogeneity (172, 173). Complete control of OC will likely require simultaneous targeting of more than one feature.

In addition to CA-125, several therapeutics targeting NK cells to specific OC-associated antigens to activate ADCC are emerging. These include monoclonal and bi-specific antibodies, antibody-drug conjugates alone, and in combination with CAR-engineered NK cells (165, 174). One notable study found that the bispecific monoclonal antibody anti-EpCAM x anti-CD3 simultaneously activated T and NK cells with a strong enough interaction to overcome the immunosuppressive TME found in malignant ascites (175). Antibody-drug conjugates have also been developed for OC patients (176). Of these, Mirvetuximab Soravtasine (IMGN83), an anti-folate receptor alpha antibody conjugated to a cytotoxic maytansinoid, is the most developed and has demonstrated efficacy as a single agent *in vivo* (177). Unfortunately, this efficacy did not translate into clinical efficacy: a phase III study in platinum resistant OC patients concluded that progression free survival was not superior to standard chemotherapy (178, 179). Limited efficacy in antibody-based therapies, including IMGN83, may be due to one or several strategies including acquired resistance and/or an insufficient immune response.

NK cells from cancer patients often exhibit downregulation of the Fc receptor CD16 required for ADCC, resulting in reduced efficacy of antibody-based approaches (180, 181). To overcome this challenge and augment NK cell-mediated ADCC, researchers are inhibiting the metalloproteinases that cleave CD16 (182), or specifically engineering NK cells or cell lines for permanent expression of CD16 to facilitate ADCC (183).

The combination of antigen-agnostic reactivity (i.e., through germline-encoded activating receptors) together with the abilty to recruit NK cells for responsiveness against specific tumor antigens (i.e., through CAR or antibodies) may provide the required heterogeneous immune response required to combat the highly heterogeneous cancer that is OC. Cancer phenotyping to understand this heterogeneity—with extensions in predicting the ideal configuration of NK immunity—would assist in developing more precise approaches to OC immunotherapy.

## Future Recommendations

Immunotherapies that focus and predict the specific ligands and ligand combinations for NK cells are likely to enhance OC clearance and control. NK cells can provide a multifaceted approach to meet the challenge of heterogeneity in OC tumors. That NK cells can serve both antigen-specific (i.e., ADCC, CAR) and antigen-agnostic roles (i.e., DNA damage, cell stress) (15, 184), in detecting and eliminating tumors is a strength of NK-based immunotherapies, especially against the highly-heterogeneous OC.

Enhancing or replacing NK cell function in OC is both a feasible and logical strategy that complements several existing immunotherapies. To maximize NK function and activation within the TME, further research is warranted to first identify the relevance of NK cells for the outcome of OC patients. Toward the inclusion of NK cells as key players in immunotherapy we have synthesized the following recommendations for those researching how the OC TME interacts with cancer therapies. These recommendations should be considered when developing fundamental, translational and clinical studies to contribute to the growing body of knowledge surrounding NK cell relevance in OC.

1. NK cells as outcome measures
  It is crucial that NK cells be included as outcome measures in clinical trials evaluating immunotherapies. For example, anti-PD-1 therapies characterize T cell populations and neglect the evaluation of other lymphocytes. Likewise, NK cells are important mediators of ADCC; therefore, NK cell function should be considered in antibody-based approaches.
2. Further characterization of NK cells in OC
  Identifying phenotypic classification and prognostic value of NK in OC may also aid in the stratification of patients for therapy. For example, identifying KIR immunogenomic status may allow identification of immunotherapeutic responders from non-responders based on immunogenetics and NK cell licensing. Inspiration for this can be drawn from investigations in other cancers that have shown important contributions of NK cell education and inhibition in contributing to therapeutic outcomes (19).
3. Measure leukocyte reactivity and compositions in the TME
  Efficient tumor control by immune mechanisms involves the collaboration of innate and adaptive lymphocytes. Thus, strategies to better understand immune function in the tumor will identify shortcomings in the current approaches and keys to next-generation immunotherapies.

## Conclusions

Limited treatment options, no effective screening strategies, high recurrence, and poor overall survival emphasize the need for improving therapeutic strategies to combat OC. It is clear that current treatments are unable to control OC progression. As with several other hard-to-treat tumors, researchers and oncologists are turning to immunotherapy to treat OC. Immune cell infiltration carries both predictive and prognostic value in OC; however, the complex relationship between the immune system and tumor remains a topic of active study. This investigation has revealed an extremely immunosuppressive TME in OC that results in the dysregulation of immune effector cells leading to immune evasion and tumor progression.

While immunotherapies have encountered several challenges, strategies are being developed to improve their efficacy. Cytokine treatments now focus on enhancing specificity and safety. The development of cytokine superagonists and oncolytic virus delivery strategies can theoretically provide the required specificity to bring these into clinical use. Similarly, adoptive cell transfer therapies are being enhanced to establish feasibility and efficacy. Most promising are combination immunotherapies designed to target and activate multiple immune pathways.

These advances demonstrate promise to improve immunotherapies and minimize associated toxicities; however, without strategies to efficiently identify OC responders, immunotherapies will continue to yield disappointing results. The ability to properly stratify patients relies on understanding of a patient's underlying immunity and the pre-existing immune TME. It also requires an in depth understanding into how these factors interact with and respond to chemo-, radio- and immuno-therapies. By focusing and targeting immunotherapies to individual pathways, including those which enhance functionality of just one cell type or one immune cell pathway, the therapeutic impact may be limited. The broad functions of NK cells make them amenable for immunotherapy because they can mediate tumor killing using a variety of mechanisms, including ADCC and germline-encoded receptors, with minimal toxicity. Alone, or in combination with existing strategies, NK cells hold great promise for treatment of OC.

## Author Contributions

JB and SN conceptualized the review. SN, HG, JT, SG, and JB wrote and edited the review. SN, HG, and SG generated the tables.

### Conflict of Interest Statement

The authors declare that the research was conducted in the absence of any commercial or financial relationships that could be construed as a potential conflict of interest.
